# Sex-specific differences and how to handle them in early psoriatic arthritis

**DOI:** 10.1186/s13075-021-02680-y

**Published:** 2022-01-11

**Authors:** E. Passia, M. Vis, L. C. Coates, A. Soni, I. Tchetverikov, A. H. Gerards, M. R. Kok, P. A. J. M. Vos, L. Korswagen, F. Fodili, Y. P. M. Goekoop-Ruiterman, J. van der Kaap, M. van Oosterhout, J. J. Luime

**Affiliations:** 1 Department of Rheumatology, Erasmus University MC, NB 850, PO box 2040, 3315EJ Rotterdam, The Netherlands; 2 Nuffield Department of Orthopedics, Rheumatology and Musculoskeletal Sciences, Un. of Oxford, Oxford, UK; 3 Albert Schweitzer H., Dordrecht, The Netherlands; 4 Fransicus H., Rotterdam, The Netherlands; 5 Maasstad H., Rotterdam, The Netherlands; 6 Amphia H., Breda, The Netherlands; 7 Reumazorg Zuid West Nederland, Roosendaal, The Netherlands; 8 Haga H., Den Haag, Hague, The Netherlands; 9 Groene Hart H., Gouda, The Netherlands

**Keywords:** Psoriatic arthritis, Sex differences

## Abstract

**Objectives:**

The prevalence of psoriatic arthritis (PsA) is the same in men and women; however, the latter experience a higher burden of disease and are affected more frequently by polyarthritis. Here, we performed an early PsA cohort analysis to assess sex-related differences in demographics, disease characteristics, and evolution over 1 year including applied treatment strategies.

**Methods:**

Our study is embedded in the Dutch south-west Early Psoriatic Arthritis cohoRt. We described patient characteristics and treatment decisions. For the comparison across sexes and baseline and 1 year follow-up, appropriate tests depending on the distribution were used.

**Results:**

Two hundred seventy-three men and 294 women with no significant differences in age and ethnicity were included. Women reported significantly longer duration of symptoms before diagnosis and significantly higher tender joint count, a higher disease activity, higher levels of pain, and lower functional capacity. Although minimal disease activity (MDA) rates increased over time for both sexes, MDA remained significantly more prevalent among men at 1 year (58.1% vs 35.7%, *p < 0.00*).

Initially, treatment strategies were similar in both sexes with methotrexate being the most frequently used drug during the first year. Women received methotrexate for a shorter period [196 (93–364) vs 306 (157–365), *p < 0.00*] and therefore received a lower cumulative dose compared to men. Retention time was shorter for all DMARDs, and women had a delayed start on b-DMARDs.

**Conclusion:**

After 1 year of standard-of-care treatment, women did not surpass their baseline disadvantages. Despite the overall improvement, they still presented higher disease activity, higher levels of pain, and lower functional capacity score than men. The nature of these findings may advocate a need for sex specific adjustment of treatment strategies and evaluation in early PsA patients.

**Supplementary Information:**

The online version contains supplementary material available at 10.1186/s13075-021-02680-y.

What is already known about this subject?

- Although the prevalence of Psoriatic Arthritis is equal between men and women, the burden of disease is higher for women than for men in established disease

What does this study add?

- Differences in disease burden are present from the diagnosis onwards
- Improvement of Disease activity evolves to the same extend for the continuous measures, except for reaching Minimal Disease Activity (MDA). MDA is more often achieved by men
- DMARD frequency and dosing are initially equal, but over time cumulative doses and DMARD retention time were lower in women

How might this impact on clinical practice or future developments?

- The nature of these findings may advocate a need for sex specific adjustment of treatment strategies and evaluation in early PsA patients.

## Introduction

Sex and health is a new area of study, aiming to investigate the differences between men and women in both health and disease. Sex has been shown to affect natural history, clinical manifestations, and response to medications in several rheumatic diseases. Although the prevalence of psoriatic arthritis (PsA) is considered equal in men and women, they are not equally affected, with women experiencing a higher burden of disease (pain, disability and fatigue) [1–4]. A few studies though suggested that the prevalence of PsA is higher in men [5–7], and at the same time, others have demonstrated the opposite [8, 9]. Differences also seem to exist in clinical expression of PsA with men assembling more peripheral and axial joint impairment and women being affected more frequently by polyarthritis, higher tender joint count, and higher scores of functional disability [1–4]. Development of radiographic joint damage is more likely present in men whereas women seem to report lower quality of life [1]. Women are also more likely to present a treatment-resistant PsA disease and compared to men are reported to have higher PsA life impact [10, 11]. According to the international study of Orbai et al., the present treatment protocols seem not adequate to overpass the life impact divergence between men and women [12].

Sex differences in PsA are not yet embedded in clinical practice or in the scientific thinking as there is little knowledge about clinical expression and disease activity differences by sex in PsA patients. Additional research is needed to explore the impact of sex on clinical expression, disease burden, treatment prescription, and response in PsA, something that would improve disease management and contribute to an optimal therapy.

The objectives of our research project are to assess sex-related differences in baseline demographics, disease characteristics, and comorbidities in patients with newly diagnosed PsA and, secondly, to evaluate PsA evolution over time (1 year follow-up), stratified by sex, for disease activity and health-related quality of life. Our further objective is to identify whether there is a diversity of therapeutic decisions between sexes in our cohort in the first year after diagnosis and their relation with the aforementioned findings.

## Methods

### Patients and setting

Our study is embedded in the Dutch south-west Early Psoriatic Arthritis Registry (DEPAR) [13], a prospective cohort study which included newly diagnosed patients with PsA. The diagnosis was made by rheumatologists and based on expert opinion; no classification criteria were applied to ensure enrollment of a population representative of daily clinical practice. For this analysis, data were used from patients included between August 2013 and February 2019 recruited in centers in the southwest of the Netherlands (1 academic and 10 general hospitals, 1 treatment center specialized in rheumatic care). Patients who participated provided a written informed consent according to the Declaration of Helsinki. The local medical research ethics committee of Erasmus University Medical Center of Rotterdam authorized the study.

### Data collection

Information for newly diagnosed patients with PsA was collected every 3 months during the first year after diagnosis. DEPAR cohort’s research nurses collected clinical information, medical records, and carried out clinical examination. Research nurses are trained annually by the same rheumatologist. Joints were evaluated for pain 68 joints and swelling 66 joints according to the EULAR handbook of clinical assessments. Enthesitis scores were performed using the methods described by the authors of the LEI and MASES papers, assessing pain when applying pressure on prespecified entheseal points. Axial involvement was assessed using clinical assessment: New York criteria for inflammatory back pain and the BASDAI.

Patients also completed a broad spectrum of patient-reported outcome measures (PROMs) before each appointment with nurses.

### Patient reported outcomes and disease activity measures

We evaluated the health status and the impact of PsA to the patients with newly diagnosed PsA using patient’s global and pain scores on a visual analog scale (VAS), fatigue using the Bristol Rheumatoid Arthritis Fatigue (BRAF) questionnaire [14], health-related quality of life (HRQoL) by the Hospital Anxiety and Depression Scale (HADS) [15], and the Short Form 36 Health Survey (SF-36) presented by the Physical Component Summary (PCS) and Mental Component Summary (MCS) [16]. The functional status of the PsA patients was evaluated using the Health Assessment Questionnaire (HAQ) [17]. We evaluated the skin disease of the patients who presented concomitant PsA and psoriasis using the Psoriasis Area and Severity Index (PASI) score [18] and the impact using the Skindex-17 questionnaire [19].

We measured disease activity using activity parameters commonly used in clinical practice and research [20] for patients diagnosed with PsA: minimal disease activity (MDA) [21], GRAppa Composite ScorE (GRACE) [22], Psoriatic Arthritis Disease Activity Score (PASDAS) [23], Disease Activity Psoriatic Arthritis) (DAPSA), and Composite Psoriatic Disease Activity Index (CPDAI) [24].

### Statistical analysis

Patient characteristics are described using simple descriptive analysis techniques, with continuous variables summarized by their means (and SD) or medians (and IQR) and categorical variables summarized by their proportions. For the unadjusted comparison across men and women, Pearson’s chi-squared test was used for categorical variables, and *t* test was used to compare continuous variables that are normally distributed, whereas Kruskal-Wallis was used for continuous variables that are not normally distributed. A *p* < 0.05 is considered statistically significant.

Comparisons at baseline and at 1-year follow-up and between subgroups (men, women) were performed with Pearson’s chi-squared test and Kruskal-Wallis test. Difference in evolution over time of continuous variables between the sexes was tested by difference in slope using the partial derivative of the response with respect to time separately by sex of a linear mixed models (LMM) in which time and sex interacted. Binary measures were analyzed using logistic mixed models to account for repeated measures over time (MDA). Five time points were used: baseline, 3, 6, 9, and 12 months after diagnosis.

Simple descriptive analysis techniques were also used in order to describe the therapeutic decisions and their differences between sexes. As many different strategies were used, both in type of drug and combination of drugs as well as dosing, we decided to simplify medication use. To be able to see differences in transitions of drugs over time between the sexes, we categorized the DMARDs in most potent drug categories: b-DMARDs, methotrexate, cs-DMARDs (other than methotrexate), GCs (glucocorticosteroids), and no-DMARDs. Missing values of any type of dependent or independent variables were not imputed as all patients were included from their initial moment of participating with available data independent whether they had a complete 1-year of follow-up.

Statistical analyses were performed using STATA-16.

## Results

### Sex-related differences in baseline demographics, disease characteristics, and comorbidities between men and women with newly diagnosed PsA

In February 2019, out of 620 patients who participated in DEPAR cohort, a total of 307 men and 313 women were available for analysis. Both groups were similar regarding ethnicity and smoking. There were no differences across men and women in the age at onset of PsA; however, women reported longer duration of symptoms before diagnosis of PsA (11 vs 7.4 months, *p < 0.00*). Years of daytime education as proxy for educational level was 1 year (*p < 0.05*) less in women (12; IQR 10–15) compared to men (13; IQR 11–16) and fewer women than men were in paid employment (64% vs 78%, *p < 0.00*). The prevalence of obesity (BMI > 30 kg/m^2^) was higher in women than in men (36% versus 28%, *p < 0.05*).

**Fig. 1:**
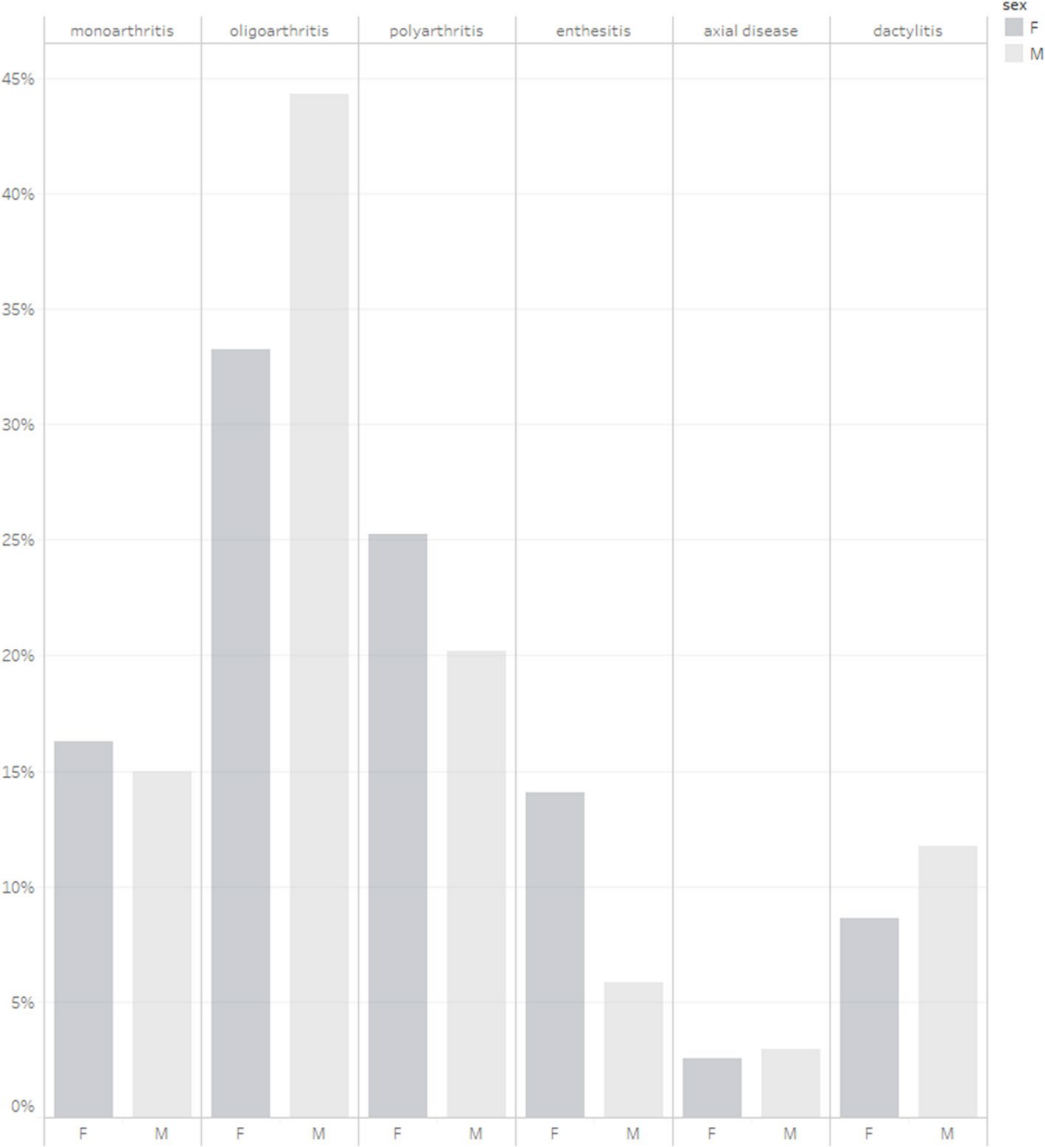
Phenotypes at Baseline by Sex

Oligoarthritis was the predominant pattern of arthritis in both men and women but with a significantly higher prevalence in men (45.9% in men vs 34.2% in women, *p < 0.05*). Polyarthritis was more prevalent in women (25.4% in women versus 19.6% in men), but this difference was not statistically significant. Clinically defined enthesitis had a significantly higher prevalence in women than in men (14.3% vs 5.9% in men, *p < 0.05*). There was a significantly higher proportion of women with axial disease as first manifestation of PsA; however, the small sample size available may affect the reliability of this result (Fig. 1).

Women presented at baseline higher tender joint count than men (TJC68: 4 vs 2, *p < 0.00*), whereas there was no difference in swollen joint count (SJC66). All composite indices studied (CPDAI, DAPSA, GRACE, MDA, PASDAS) showed significantly worse results in women at baseline (Table 1). There was no statistically significant difference of absolute CRP levels although CRP was positive (> 5 mg/L) in 41% of men and 30% of women (*p < 0.05*).

**Table 1:** Patient characteristics at baseline by sex

	All	Men	Women	*p* < 0.05
	*n* = 620	*n* = 307	*n* = 313	
Age at onset of PsA*	50 (39–60)	50 (40–61)	51 (38–60)	
Duration of symptoms (months)*	11 (3.6–33.3)	7.4 (3.2–25.6)	13.5 (4.6–44.8)	*
Working status (age < 68), yes**	71%	78%	64%	*
Educational status (years)*	12 (10–15)	13 (11–16)	12 (10–15)	*
Ethnicity (Dutch)**	94%	94%	93%	
Smoking, yes**	21%	18%	24%	
BMI*	27.5 (25–31.4)	27.2 (24.8–31.1)	27.9 (24–31.7)	
Obesity (BMI > 30 kg/m^2^)	32%	28%	36%	*
CRP (mg/L)*	3.9 (0–10)	4 (0.8–11)	3.4 (0–9)	
CRP positive (> 5 mg/L)	36%	41%	30%	*
SJC66*	2 (1–4)	2 (1–4)	2 (0–4)	*
TJC68*	3 (1–7)	2 (1–5)	4 (2–8)	*
VAS global*	47 (24–65)	40 (19–58)	51 (31–70)	*
Pain (VAS score)*	47 (25–69)	41 (21.5–62)	53 (31–71)	*
PASI*	2 (0.4–4.4)	2.4 (0.6–5.2)	1.45 (0.3–3.45)	*
Anxiety**	4 (2–7)	4 (1–6)	5 (3–8)	*
BRAF*	20 (10–31)	16 (6–27)	25 (15–34)	*
HAQ*	0.75 (0.38–1.13)	0.63 (0.19–0.88)	0.88 (0.50–1.25)	*
CPDAI*	4 (2–5)	3 (2–5)	4 (3–6)	*
DAPSA*	21 (13–32)	19 (11–32)	22 (15–32)	*
GRACE*	3.4 (2.3–4.3)	3.0(2.0–4.3)	3.6(2.6–4.5)	
MDA**	31%	30%	32%	
PASDAS***	4.2 (3.3–4.9)	4 (3.1–4.8)	4.3 (3.5–5)	*

BMI, body mass index, BRAF, Bristol Rheumatoid Arthritis Fatigue; CPDAI, Composite Psoriatic Disease Activity Index; CRP, C-reactive protein; DAPSA, Disease activity index for Psoriatic Arthritis; GRACE, GRAppa Composite ScorE; HAQ, Health Assessment Questionnaire; IQR, interquartile range; PASDAS , Psoriatic Arthritis Disease Activity Score; PASI, Psoriasis Area and Severity Index; SJC66, swollen joint count; TJC68 , tender joint count; VAS, visual analog scale. A *p* < 0.05 is considered statistically significant

*median (IQR)

**n(%)

***mean (SD)

Skin lesions were more prevalent in men. Although women were more likely to report a positive family history of psoriasis, men presented significantly higher PASI score [2.4 (IQR 0.6–5.2) versus 1.5 (IQR 0.3–3.5) in women.

Women presented more frequently fatigue, anxiety, and comorbid medical conditions (chronic inflammatory diseases). At baseline, women suffered more severe limitations in function and worse quality of life compared with men based in all patients’ reported outcomes (Table 1).

### PsA evolution over 1 year of follow-up, stratified by sex

Among the 108 patients (19% of the DEPAR population) that did not yet complete the 12 month follow-up or dropped out, 53 (19.4% of men population) were men and 55 (18.7% of women population) were women. Overall, there were no statistically significant differences between sexes concerning the reason and the timing of discontinuation before T12 (Supplementary Table 1 a,b).

**Fig. 2:**
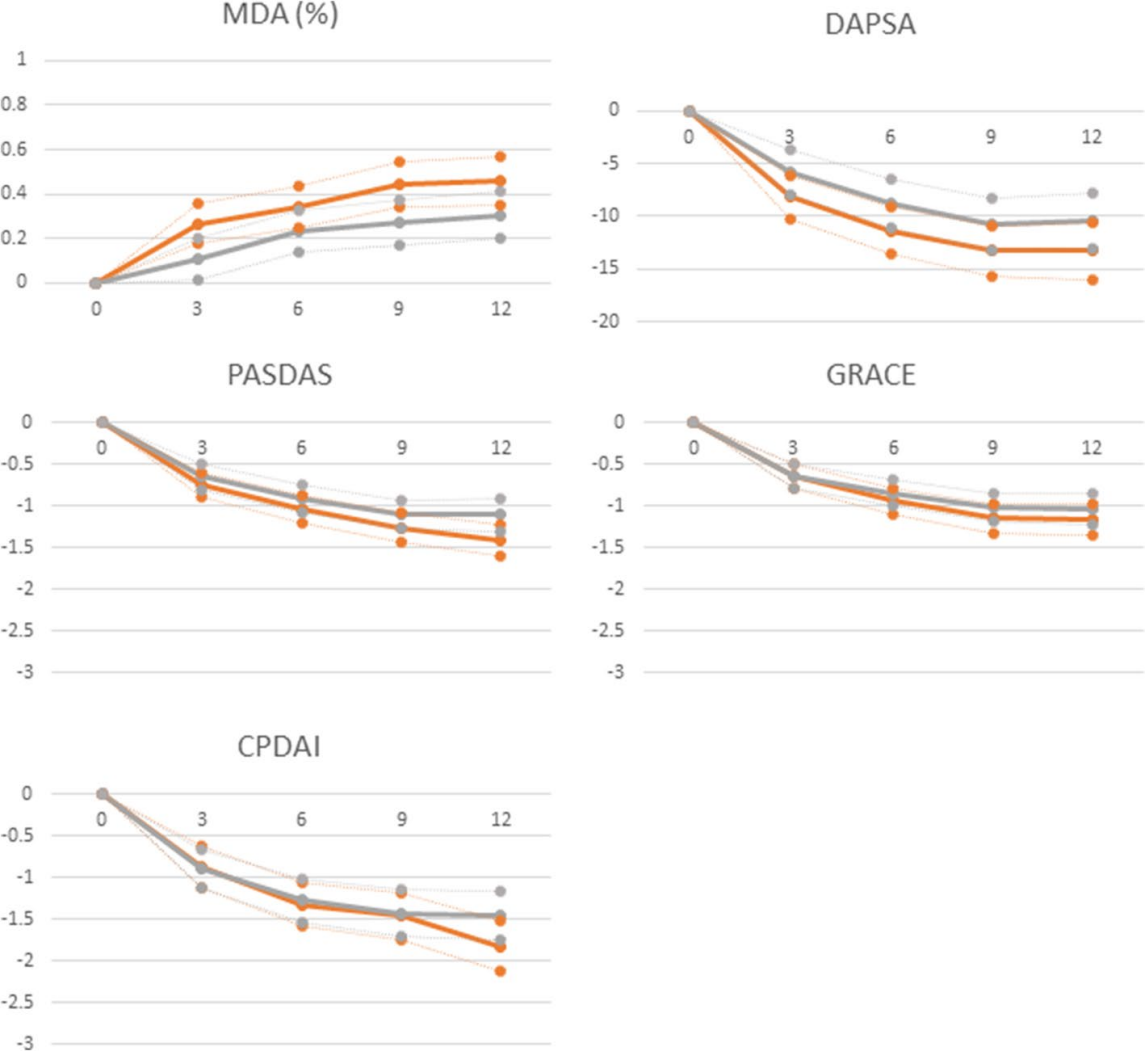
Comparison of evolution of disease activity over time between de sexes as expressed in mean change from baseline

The composite disease activity measures studied (CPDAI, GRACE, PASDAS, DAPSA) presented also similar pattern of evolution through the year for both men and women (Fig. 2); however, they were significantly higher in women at both time points (Tables 1 and 2 and Supplementary Figure 2). MDA, although its progressive beneficial change through time for both sexes (Fig. 2), remained predominantly present among men (18.0% vs 10% at inclusion, *p < 0.05*, and 59% vs 37%, *p < 0.00*, at 1-year follow-up). Furthermore, DAPSA remission, the other treatment target in PsA [24], showed a significantly higher percentage of men (28% vs 11% of women, *p < 0.00*) presented remission according to DAPSA (⩽4) at 12 months of follow-up.

**Table 2:** Patient characteristics stratified on sex at 1-year follow-up (T12)

Characteristic	1-year follow-up		
	Men (*n* = 220)	Women (*n* = 233)	*p* < 0.05
**Laboratory values**			
CRP	2 (0–4.9)	2 (0–5)	#
CRP > 5 mg (%)	13	16	
**Clinical assessment**			
TJC 68	0 (0–2)	1 (0–4)	#
SJC 66	0 (0–1)	0 (0–1)	
PASI	1.2 (0–2.8)	0.45 (0–1.8)	#
**Patient questionnaire**			
VAS global	18 (5–37)	29 (12–51)	#
VAS pain	16 (4.5–40)	31 (12–60)	#
HAQ	0.13 (0–0.63)	0.75 (0.25–1.1)	#
Skindex-17—symptoms	3 (1–4)	3 (1–5)	#
Skindex-17—psychosocial	0 (0–3)	0 (0–3)	#
SF36-PCS	48 (39–53)	42 (35–47)	#
SF36-MCS	54 (46–59)	48 (40–55)	#
BRAF	13 (6–22)	22 (15–32)	#
**Composite measures**			
CPDAI	1 (1–2)	2 (1–4)	#
DAPSA	7.5 (3.3–15)	13 (6.8–22)	#
GRACE	1.4 (0.6–2.7)	2.3 (1.3–3.6)	
MDA, yes (%)	59	37	#
PASDAS	2.2 (1.6–3.3)	3 (2.1–4)	#

^#^*p* < 0.05, Fischer’s exact test

Swollen joint count showed statistically significant improvement in both men and women at 1 year follow-up (Table 2). Both groups presented improvement of CRP at the end of the 1st year. Interestingly, the percentage of women with positive CRP (CRP > 5 mg/l) remained high at 1 year follow-up (28.8% at inclusion and 21.4% at 1 year follow-up, *p* = 0.06).

Women, despite the improvement that they presented through 1 year follow-up, reported higher levels of pain (VAS) compared to men at 1 year follow-up (16 vs 31, *p < 0.00*). Similarly, HAQ improved through time for both men and women but remained statistically higher in the latter (Table 2). BRAF score (corresponding to fatigue) shows a limited beneficial change through time for both men and women (Table 2).

### Therapeutic decisions in DEPAR cohort, by sex

At baseline, 54% of the women and 56% of the men started with methotrexate, 12% of the women and 6% of men started another cs-DMARD, while 2% of the women and 6% of the men used an b-DMARD. The use of b-DMARDs in the Netherlands is allowed after failure on a cs-DMARD as established by a rheumatologist with no formal requirements on definition of treatment failure. Early use or start of b-DMARDs in DEPAR was either due to patients already using them, axial disease, or the prior use of methotrexate in psoriasis that did not prevent onset of arthritis. In 32% of the females and 33% of the men no DMARD was prescribed, while 1% of the women and 2% of the men used GCs only.

Over time, different treatment strategies were followed as shown in Table 3. Here, we present the most remarkable differences in the frequency of the most dominant DMARD and also in total exposure to the different DMARDs expressed in cumulative doses for the cs-DMARDs and in number of days for the b-DMARDs. After 3 months, 77% of the women retained their methotrexate as dominant drug, 10% of the women refrained from any DMARD, 6% was changed to another cs-DMARD, 6% to a b-DMARD, while another 1% only used GCs. This compared to men of who 88% kept their methotrexate, 7% changed to no-DMARDs, 4% to another cs-DMARD and 1% to a biological. Over the subsequent 3-month intervals of the follow-up, the patterns of change became more comparable between men and women with most patients staying in the same drug category as they were already in. At 12 months the use of methotrexate had decreased in both sexes to 41%, while no-DMARD use had increased to 34%. b-DMARD use increased to 13% and another 13% of the women and 10% of the men used cs-DMARDs.

**Table 3:** Dominant drug type and transition of drug type over 3 months intervals stratified for women and men

Timepoint	Previous DMARD use	DMARD use									
		Women					Men				
		Methotrexate	Steroids	cs-DMARDs	b-DMARDs	no DMARD	Methotrexate	Steroids	Cs-DMARDs	b-DMARDs	None
**Baseline**		54%	1%	12%	2%	32%	56%	2%	6%	4%	33%
**T3**	**Methotrexate**	77%	1%	6%	6%	10%	88%		4%	1%	7%
	**Steroids**	50%	50%				40%	40%	20%		
	**cs-DMARDs**	17%		63%		20%	18%		65%		18%
	**b-DMARDs**				100%					91%	9%
	**None**	13%		4%		84%	11%		1%	1%	87%
**T6**	**Methotrexate**	88%		5%	3%	4%	82%	1%	2%	6%	9%
	**Steroids**	50%	50%					100%			
	**cs-DMARDs**	13%		81%		6%	5%		79%	5%	11%
	**b-DMARDs**	13%			73%	13%			7%	79%	14%
	**None**	10%		5%	3%	81%	9%	1%	1%	1%	87%
**T9**	**Methotrexate**	69%		12%	6%	13%	78%		5%	6%	12%
	**Steroids**		100%				25%	25%	25%		25%
	**cs-DMARDs**	11%		78%		11%			72%	11%	17%
	**b-DMARDs**	22%			72%	6%	5%			79%	16%
	**None**	7%		2%	5%	87%	4%		3%		93%
**T12**	**Methotrexate**	79%		3%	7%	11%	83%	1%	6%	3%	7%
	**Steroids**			100%				100%			
	**cs-DMARDs**	15%		56%	15%	15%	6%		67%	11%	17%
	**b-DMARDs**	5%			86%	10%	4%			85%	12%
	**None**	13%		6%	1%	80%	12%	1%	5%	1%	80%

*cs-DMARD, conventional synthetic DMARDS excluding methotrexate

Cumulative doses and drug survival/retention time differed between men and women in first year after diagnosis; see Table 4. Our analysis showed no statistically significant differences between sexes concerning the 1st dose of the 1st prescribed treatment. However, when analyzing cumulative doses and total period of the prescribed treatment, we observed a statistically significant difference between sexes regarding the cumulative dose of methotrexate. More specifically, during the first year after diagnosis, men received a higher cumulative dose of methotrexate (757 mg vs 543 mg, men versus women respectively, *p < 0.00*) and for a prolonged period of time (306 days (♂) vs 196 days (♀); *p < 0.00*). Similar results were observed for the prescription of methotrexate subcutaneous injection (557 mg (♂) vs 365 mg (♀) *p* < 0.00 and 183 days (♂) vs 166 days (♀) *p* < 0.05). Women treated with sulfasalazine received also lower cumulative dose and for a shorter period of time compared to men [(548 gr (♂) vs 216 gr, *p < 0.00* and 276 (♂) vs 108 days (♀), *p < 0.*00)]. We observed no differences in daily or weekly dose for the above mentioned DMARDs, neither did we for the cumulative dose and/or duration of prescription of the other cs-DMARDs or glucocorticoids. For the b-DMARDs, we saw an earlier start for men compared to women and, likely due to that, a lower number of days exposed for women although this was not statistically significant.

**Table 4:** Days, cumulative dose and dose of DMARDs the 1st year after diagnosis stratified for men and women

		Men (*n* = 304)				Women (*n* = 311)				
		*n*	p25	p50	p75	*n*	p25	p50	p75	*p* value*
Biological	Days	42	106	212	319	39	83	160	260	0.10
Methotrexate	Days	216	208	354	365	215	174	336	365	0.05^t^
	Cumulative dose (mg)		541	809	1172		423	770	1132	0.03^t^
	Dose		15	20	24		15	20	24	0.14^t^
Sulfasalazine	Days	36	149	289	319	52	43	97	277	0.002
	Cumulative dose (mg)		258000	549500	614000		34250	174000	433000	0.001
	Dose		1741	1931	2000		1000	1925	2000	0.35
Hydroxychloroquine	Days	11	24	173	289	34	116	270	344	0.13
	Cumulative dose (mg)		9600	69200	115600		39600	88600	137600	0.30
	Dose		400	400	400		400	400	400	0.54
Leflunomide	Days	22	100	197	281	37	50	126	212	0.06
	Cumulative dose (mg)		1740	2610	4940		900	2020	2775	0.07
	Dose		10	20	20		10	17	20	0.59
Prednisone	Days	32	34	98	171	51	38	89	183	0.66
	Cumulative dose (mg)		648	1208	1820		420	814	1480	0.20
	Dose		8	11	20		7	8	15	0.06

*Non-parametric comparison between men and women

^t^Parametric *t* test for comparison between men and women

DMARD side-effects were reported for 198 patients, more for women (58%) than for men (42%). About 38% were reported in the first 3 months, followed by 26% to 17% in the subsequent 3-month intervals. Methotrexate was most frequently mentioned with 84%, followed by sulfasalazine, 16%, and leflunomide, 9%. Both sexes experienced equally side-effects for methotrexate and adalimumab, but side-effects for the other drugs were more often reported by women.

## Discussion

Symptoms, disease presentation, and treatment strategies in early PsA differed between men and women in the first year of disease. Women reported longer duration of symptoms before diagnosis, and fewer of them were in paid employment. Oligoarthritis was the predominant pattern of arthritis in both sexes. Polyarthritis and enthesitis were more prevalent in women who also presented at baseline with a higher tender joint count than men, but no difference in swollen joint count. With the exception of DAS28CRP, all composite indices (CPDAI, DAPSA, GRACE, MDA, PASDAS) showed significantly worse results in women. Cumulative doses and DMARD retention time differed between men and women in first year after diagnosis, although there were no differences in 1st dose of the 1st prescribed treatment. Women were more often switched to cs-DMARDs, and there was a tendency delayed start on b-DMARDs, although not statistically significant.

In our study, we observed that women reported higher levels of pain at the time of diagnosis but also, despite the improvement, the levels of pain reported in women remained higher compared to men at the end of one year of follow-up. Population-based research in pain has shown that women report pain more frequently than men, while studies using experimentally induced pain models demonstrate increased pain sensitivity to painful stimuli in women [25]. It is worrisome that women reporting more pain than men may contribute to underdiagnosis or late recognition of symptoms in women, influence management decisions, and introduce sex bias in prescribing. Therefore, pain reported by women should receive specific attention by attending physicians. Furthermore, the physicians should be aware of pain being reported differently by man and woman in disease management, since most disease activity measures contain pain and quality of life measurement metrics that may perform differently by sex. Also, the impact of pain on disability indices may be less pronounced in men due to their greater muscle strength, making it easier to perform daily tasks. Muscle strength has been shown to significantly affect the HAQ in patients with rheumatoid arthritis (RA) [26, 27]. That being said, the differences between men and women observed are not only related with differences in pain but could also be related with differences in underlying inflammation, hormonal changes, genetic, or other factors, physical activity that need additional research.

In our study, we observed that initially no different treatment regimens were followed across sexes; however, for specific drugs (methotrexate, sulfasalazine, adalimumab), retention time and cumulative doses were lower in women despite the higher disease burden observed in the later at diagnosis. The fact that women reported more frequently side effects compared to men could be a potential explication for the above-mentioned differences in prescription.

Overall, women in the DEPAR cohort presented higher disease activity, pain, and functional impairment compared to men at baseline but also at 1 year of follow-up. Although similar therapeutic approaches in men and women are followed, in some cases, women seem to remain undertreated. The nature of these findings could suggest that sex bias in prescribing exists and may advocate the need for sex specific adjustment of treatment strategies and evaluation of PsA.

Strengths of our study consist the large population with early PsA included in our cohort, well-recorded medication use, and the regular follow-up visits. Only a few studies have focused on early PsA and, to our knowledge, our study is the only one that focuses in sex specific differences in early PsA.

Our study has some limitations. The assessments used in order to detect the differences between sexes were a mix of objective (CRP/SJC), semi-objective (disease activity measures), and subjective tools (e.g., pain score, fatigue). More objective assessments such as MRI or radiographic findings would be useful in order to explain the observed differences between sexes. We were not able to distinguish other conditions that may impact pain and function and are not associated with PsA (e.g., fibromyalgia).

Further studies are required to assess the evolution of these differences over time but also identify the underlying mechanisms (e.g., genetic, hormonal or others) that lead to the observed divergence between men and women with newly diagnosed PsA. Moreover, further research is required to analyze the role of sex on treatment response to PsA and develop treatment strategies to improve PsA management for both sexes in daily clinical practice.

## Conclusion

After 1 year of follow-up standard-of-care treatment, women did not surpass their baseline disadvantages, and despite the improvement, they still present higher disease activity, higher levels of pain, and lower functional capacity score than men. The nature of these findings may advocate a need for sex specific adjustment of treatment strategies and evaluation in early PsA patients.

## Supplementary Information

**Additional file 1.**

## Data Availability

We are open to collaboration using DEPAR data for scientific purposes in line with the primary purpose of our cohort. To do so, please send a request to the corresponding author, briefly describing the research question and data elements you would like to use. Our scientific committee determines whether the request will be granted.

## Abbreviations

CPDAI: Composite Psoriatic Disease Activity Index
CRP: C-reactive protein
DAPSA: Disease Activity index for Psoriatic Arthritis
DAS28: Disease Activity Score 28
DEPAR: Dutch south-west Early Psoriatic Arthritis cohoRt
ESR: Erythrocyte sedimentation rate
GRACE: GRAppa Composite ScorE
HAQ: Health Assessment Questionnaire
IQR: Interquartile range
LMM: Linear mixed model
MDA: Minimal disease activity
PsA: Psoriatic arthritis
SD: Standard deviation
PASDAS: Psoriatic Arthritis Disease Activity Score
PASI: Psoriasis Area and Severity Index
SF36MCS: Short Form 36 Mental Component Scale
SJC66: Swollen joint count of 66 joints
TJC68: Tender joint count of 68 joints
VAS: Visual analog scale
