# Supplementary material for: Sex-specific differences and how to handle them in early psoriatic arthritis
Source: Arthritis Res Ther. 2022 Jan 11;24:22. doi: 10.1186/s13075-021-02680-y (PMC8751248; doi:10.1186/s13075-021-02680-y)

Supplementary File

Table S1:

s1a. Patients that didn’t complete the 12 months of follow up.

| **Reason** | **DEPAR population** | **Men** | **Women** |
| --- | --- | --- | --- |
| **less than 12 months in the study** | 67 (62%) | 33 (62.3%) | 34 (61.8%) |
| **Refusing the follow up** | 29 (26.9%) | 13 (24.5%) | 16 (29.1%) |
| **Lost of follow up** | 8 (7.4%) | 4 (7.6%) | 4 (7.3%) |
| **Moved outside** | 4 (3.7%) | 3 (5.7%) | 1 (18.8%) |
| **Total** | 108 (100.0%) | 53 (100.0%) | 55 (100.0%) |

s1b. Moment of discontinuation (patients that didn’t complete 12 months of follow up).

| **Moment of discontinuation** | **All** | **Men** | **Women** |
| --- | --- | --- | --- |
| **T0** | 22 (20.4%) | 11 (20.8%) | 11 (20.0%) |
| **T3** | 33 (30.6%) | 14 (26.4%) | 19 (24.6%) |
| **T6** | 22 (20.4%) | 11 (20.8%) | 11 (20.0%) |
| **T9** | 31 (28.7%) | 17 (32.1%) | 14 (25.5%) |
| **Total** | 108 (100.0%) | 53 (100.0%) | 55 (100.0%) |

Figure S2 : Evolution of mean disease activity measures over time stratified for sex


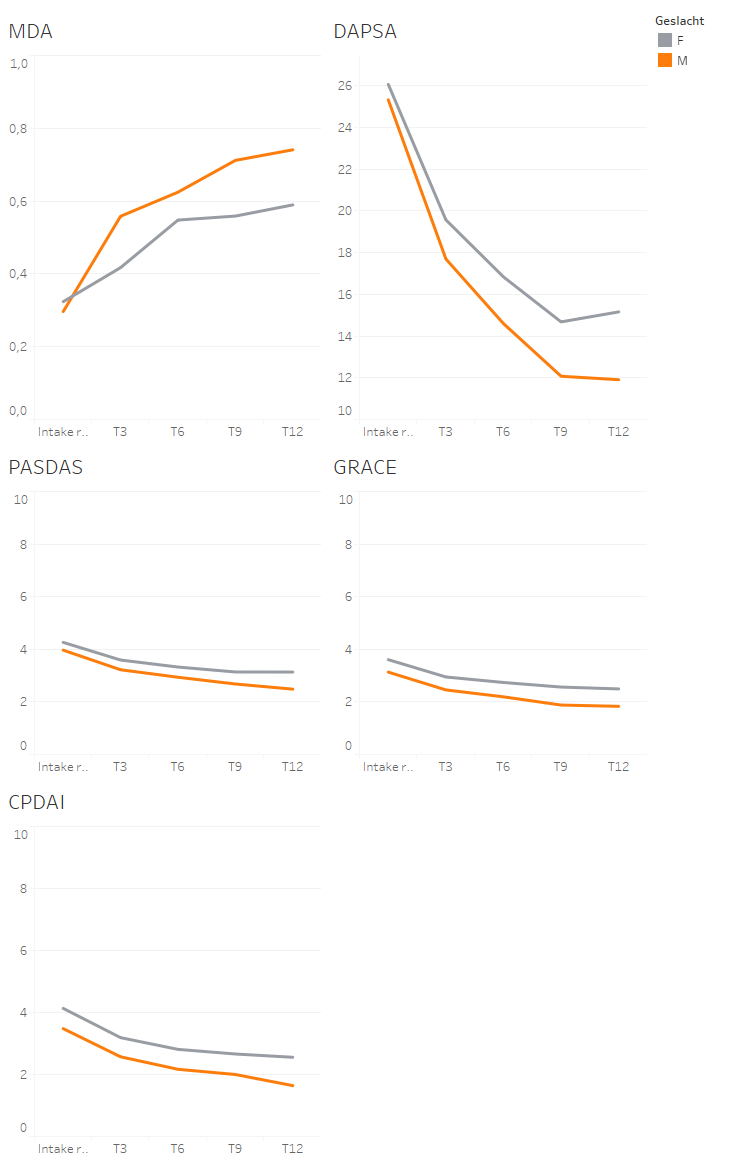

Supplement: Supplementary file 1 — Additional file 1. [file 13075_2021_2680_MOESM1_ESM.docx]
